# When to Introduce Three-Dimensional Visualization Technology into Surgical Residency: A Randomized Controlled Trial

**DOI:** 10.1007/s10916-019-1157-0

**Published:** 2019-02-09

**Authors:** Chen Lin, Junyi Gao, Hua Zheng, Jun Zhao, Hua Yang, Yue Zheng, Yihan Cao, Yufei Chen, Guoliang Wu, Guole Lin, Jianchun Yu, Hanzhong Li, Hui Pan, Quan Liao, Yupei Zhao

**Affiliations:** 1 Department of General Surgery, Peking Union Medical College Hospital (PUMCH), Chinese Academy of Medical Sciences & Peking Union Medical College (CAMS & PUMC), Beijing, China; 2 National Virtual Simulation Laboratory Education Center of Medical Sciences, PUMCH, CAMS & PUMC, Beijing, 100730 China; 3 Eight-year Program of Clinical Medicine, PUMCH, CAMS & PUMC, Beijing, China; 4 Department of Education, PUMCH, CAMS & PUMC, Beijing, 100730 China; 5 Department of Head and Neck Surgery, PUMCH, CAMS & PUMC, Beijing, 100730 China; 6 Department of Urology Surgery, PUMCH, CAMS & PUMC, Beijing, 100730 China

**Keywords:** Residency training, 3D technology, Anatomy-imaging-surgery system

## Abstract

Three-dimensional (3D) reconstructed images have been increasingly applied for medical education. Although many studies have described the benefits of such applications, the best time to introduce 3D technology into surgical training has not been determined. Therefore, we conducted a randomized study to determine a suitable period for the introduction of this technology. Seventy-one surgical residents were randomized into 2 groups (two-dimensional computed tomography (CT) group and 3D image group), and they completed a test on anatomy and imaging as well as a questionnaire. Post-graduate year 1 (PGY1) residents in the 3D group performed significantly better than those in the CT group, although the third-year residents did not present significant differences in either the score or the time spent answering the questions. Although residents in different years of training held different attitudes toward the difficulty of anatomy and imaging learning, they all showed a high level of acceptance of the 3D training. This study revealed that 3D images improved the junior residents’ performance in imaging reasoning. Thus, systematically introducing 3D images early in a surgical resident training program may help produce a better anatomy-imaging-surgery system.

**Electronic supplementary material:**

The online version of this article (10.1007/s10916-019-1157-0) contains supplementary material, which is available to authorized users.

With the rapid development of digital technologies, three-dimensional visualization systems have shown increasing potential value in medical education. A series of studies indicated that 3D anatomy models have been widely used in surgical teaching, patient education and operation planning [1–3] and that these models have many advantages over traditional methods, especially in complex anatomy, such as the larynx and inner ear [4, 5].

Standardized training of residents has become a core issue in medical education in China in recent years, and a method of enhancing the effects of surgical training within a limited time must be identified. Although many studies have suggested the benefits of combining medical teaching with 3D technologies [6, 7] a 3D-based systematic training program for residents is lacking, and few studies have described the best period for the introduction. Thus, we conducted a randomized controlled study to assess the impact of 3D reconstruction images on the identification of abnormal results and to determine the best period to introduce 3D technology into resident training programs.

## Methods

### Generating 3D simulation models with 3D technology

The 3D multi-touch visualization touchscreen table (MVT) is a large multi-touch medical table introduced by Sectra in 2010 at the Radiological Society of North America. It is approachable for medical students and surgeons for its high interactivity and short learning curve. Teachers can use the built-in cases to create real-time 3D reconstructions, and this table includes connections between the cross-sectional CT and 3D images for better medical education.

In this study, raw CT data of nine built-in cases, including two anatomical cases and seven surgical cases, were assessed, and the diseases presented in these cases included bone fracture, aneurysm, ureteral calculi, splenic rupture, pheochromocytoma and hiatal hernia. Then, 3D images were reconstructed with these CT data by the MVT. All of the 3D images were checked and assessed by two attending doctors from the surgical department of Peking Union Medical College Hospital (PUMCH), and they were used in the following tests.

### Randomized grouping

All residents participating in this study were from the surgical department of PUMCH and assigned to several groups by stratified randomization. Stratified Randomization was employed on the basis of one factor, namely post-graduate year (PGY), as this may have affected the test results (specifically PGY1, PGY2 and PGY3&4 residents constituted the stratification groups). Regarding technical details, Zelen’s algorithm was used by an invited staff from the Education Department of our center [8]. The invited staff had no conflicts of interest, did not participate further in the study and signed a Confidentiality Agreement as appropriate.

### Imaging test and questionnaire

**Fig. 1:**
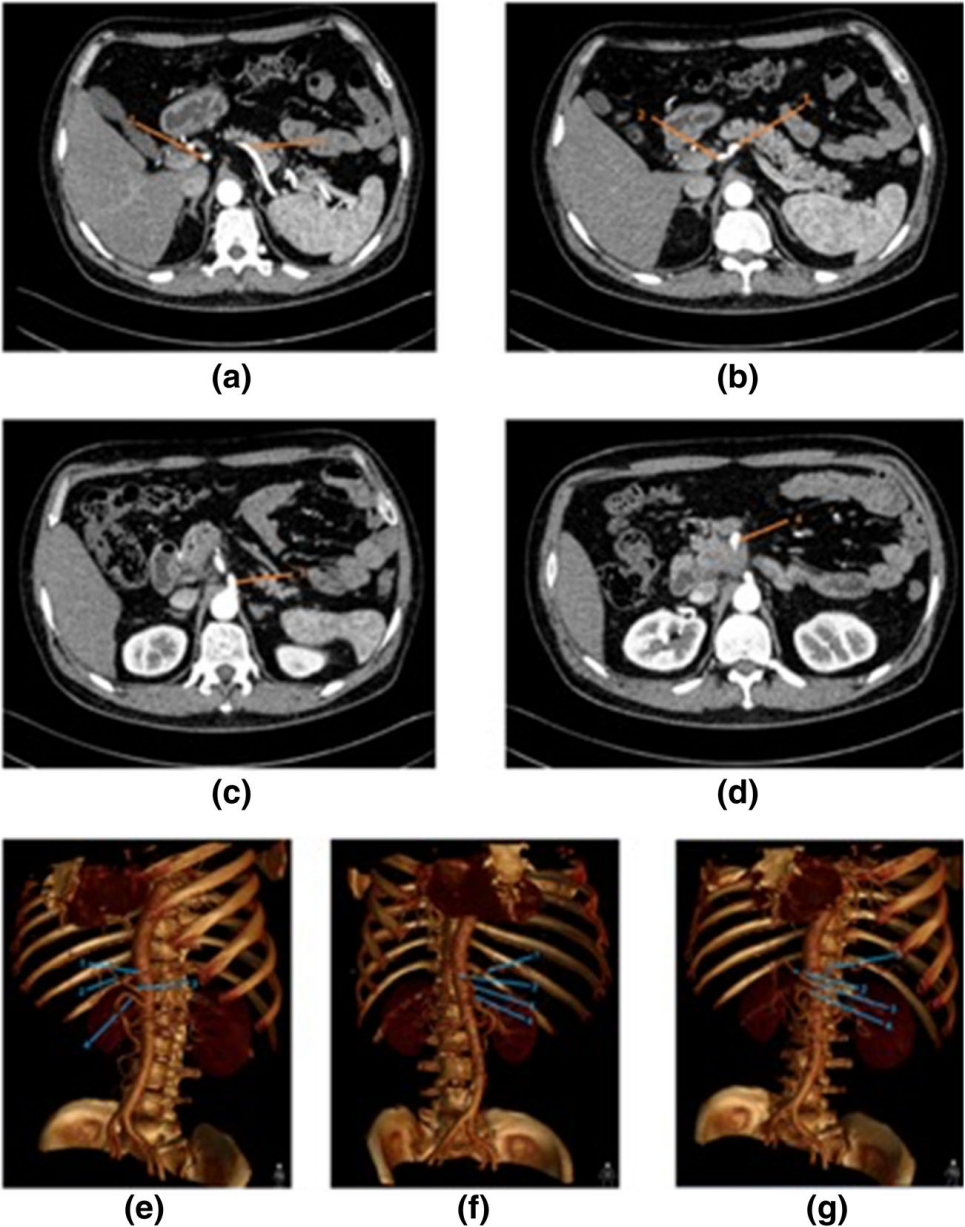
Images of an anatomy case about abdominal vessels in the CT group (a-d) and 3D group (e-g). Four points were distributed to the 1-4 anatomical structures marked with orange or blue arrows: splenic artery, common hepatic artery, celiac trunk and superior mesenteric artery

The tests consisted of 2 anatomical cases and 7 surgical cases and were developed by a group of attending doctors in the surgical field, who believe the questions are appropriate for assessing the residents’ surgical imaging reasoning abilities. The brief medical histories and questions were nearly identical for both the 3D and 2D groups except that the 3D reconstructed images and original CT images from the same cases were provided to the groups (as shown in Fig. 1).

Every case had three to four points that corresponded to detailed answers. Possible scores for the test ranged from 0 (all incorrect) to 34 (all correct). Each test paper was graded by an individual blinded to the corresponding type of images. The time each resident spent answering were also recorded by the online test system.

After the test, all residents were requested to complete a questionnaire (Table 1). This questionnaire was designed by a group of experts, based on the questionnaires of the System for the Evaluation of the Teaching Qualities (SETQ) and several studies on subjective measures toward anatomy and simulation-based education [9–11], with the aim to assess the residents’ attitudes toward anatomy learning, imaging learning and 3D training. All of these questions were rated on a 5-point Likert-type scale.

**Table 1:** Subjective evaluation questionnaire (1-strongly disagree, 5-strongly agree) and feedback results. *p*-values obtained via an independent-samples t test performed between groups of PGY1 and PGY3&4 residents

Survey Questionnaire for Medical Imaging			Feedback results			
PGY year: 1/2/3/4	Gender: Male/Female	Group type: 2D/3D	Mean scores			P*
			PGY1	PGY2	PGY3&4	
Anatomy and imaging training						
Learning approaches	1.1 A.Atlas of anatomy B.Textbooks C.Reference D.Lectures E.Videos F.3D form G.Other		–	–	–	–
Learning difficulty	1.2 It is hard to learn anatomy		4.65	4.26	3.78	<0.001
	1.3 It is hard to learn imaging reasoning		4.53	4.30	3.81	<0.001
Value for career	1.4 It is crucial to strengthen anatomy and imaging learning for a future surgical career.		4.00	4.26	4.19	0.43
3D training						
Simplification	2.1 3D images make complex anatomy easier.		3.88	3.96	4.11	0.37
Enjoyment	2.2 3D images arouse my interest in anatomy learning.		4.00	4.00	4.15	0.59
	2.3 3D images arouse my interest in imaging learning.		4.06	4.00	4.19	0.50
Intention to introduce	2.4 It is necessary to combine 3D images with 2D learning during the beginning of the residency.		4.41	4.26	4.30	0.44
Suggestions	2.5 What do you think about the 3D training? Any suggestions for future improvement? (free form)		–	–	–	–

### Statistical analysis

The summary statistics were described as the mean ± standard deviation (SD). A two-sided unpaired Student’s *t test* was applied to evaluate the influence of the 3D images on the sum scores and time spent answering, and the level of statistical significance was set at a *p* value <0.05. Categorical variables were presented as percentage. Statistical analyses were performed using SPSS (version 23.0 for Mac).

### Ethical approval

Ethical approval was obtained from the Institutional Review Board of the Institute of Basic Medical Sciences, Chinese Academy of Medical Sciences (Project No:009-2014). We maintained confidentiality by keeping relevant information in a separate database and only reviewing aggregate data. To protect the privacy of all participants, no identifying information was recorded. In addition, all of the data were stored on a password-protected computer, and the surveys were stored in a locked filing cabinet. All participants completed written informed consent. Study methods were performed in accordance with the approved guidelines.

## Results

### Characteristics of residents

In this randomized controlled study, a total of 71 surgical residents (66 males and 5 females) participated, and there was no attrition. Thirty-six residents, consisting of nine PGY1, thirteen PGY2, thirteen PGY3 and one PGY4, were randomized into the 3D group, and thirty-five residents, consisting of eight PGY1, fourteen PGY2, twelve PGY3 and one PGY4, were grouped into the CT group. The gender distribution is detailed in Table 2. In addition, the PGY4 residents were included in the PGY3&4 group because there is only two PGY4 observed in the sample.

**Table 2:** Baseline characteristics of residents

Year of residency training	Residents in the 3D CT group, no. (%)		Residents in the 2D CT group, no. (%)	
	Male	Female	Male	Female
1	8(88.9)	1(11.1)	7(87.5)	1(12.5)
2	12(92.3)	1(7.7)	14(100)	0(0)
3&4	13(92.9)	1(7.1)	12(92.3)	1(7.7)

### Sum scores of correct answers and time spent

**Fig. 2:**
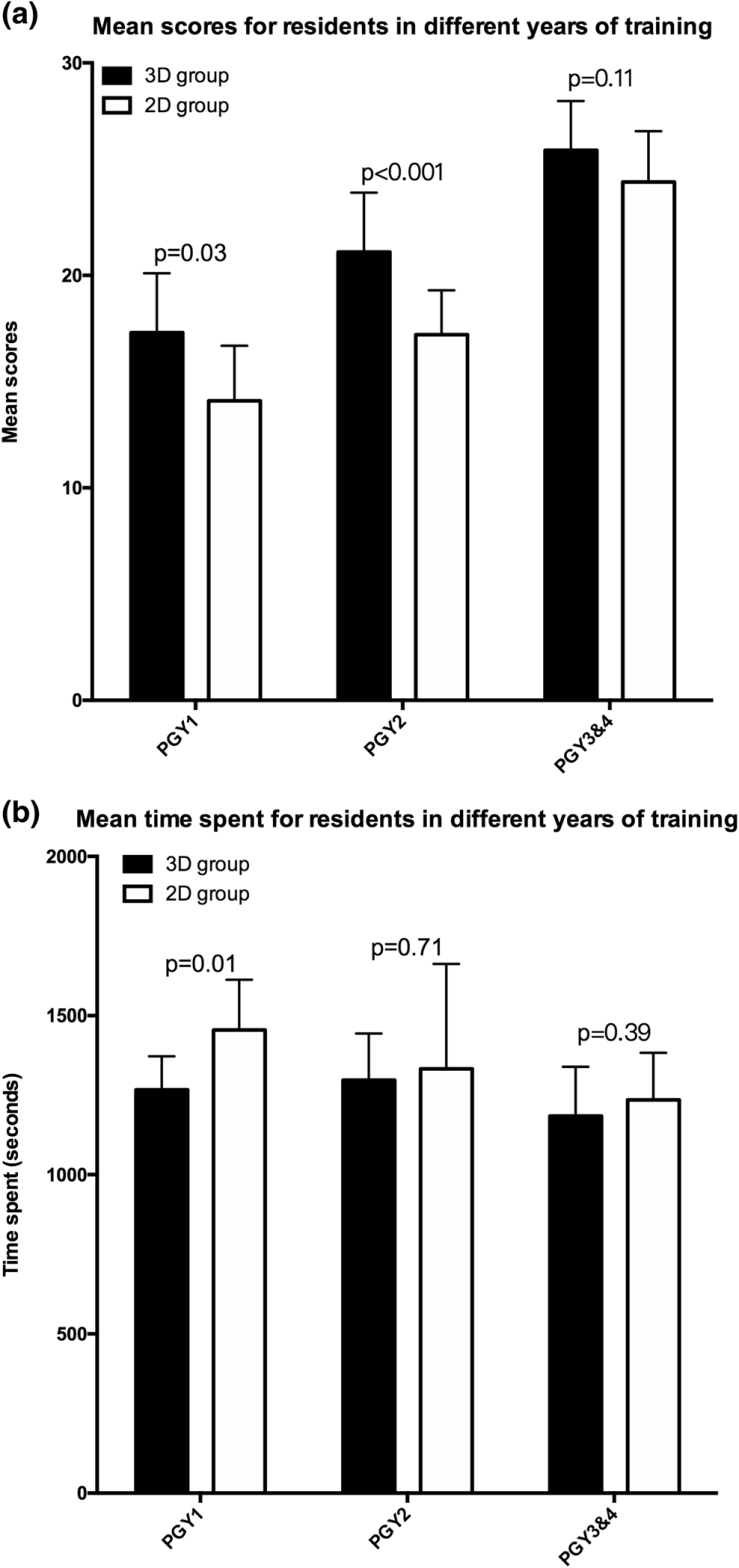
Mean scores (A) and time spent (B) for residents in different years of training. *p* value obtained via an independent-samples t-test

Overall, the PGY1 and PGY2 sum scores (Fig. 2a) were significantly different between the 3D and CT groups (PGY1, 3D vs. CT: mean difference (MD) = 3.2, *p* = 0.03. PGY2, 3D vs. CT: MD = 3.9, p<0.001), whereas significant differences were not observed in the PGY3&4 scores between the two groups (3D vs. CT: MD = 1.5, *p* = 0.11).

An analysis of the time spent answering (Fig. 2b) revealed a significant difference between the two groups of PGY1 residents; the PGY1 residents in the 3D group required 1267 ± 105 s to complete the test, whereas those in the 2D group required 1455 ± 157 s (PGY1, 3D vs. CT: MD = −188, *p* = 0.01), while there were no differences between the PGY2 and PGY3&4 residents in the two groups (PGY2, 3D vs. CT: MD = −37, *P* = 0.71. PGY3&4, 3D vs. CT: MD = −51, *p* = 0.39).

### Answers to the questionnaire

The feedback for the questions (Table 1) related to “Learning difficulty” indicated that the PGY1 residents had a more difficult time learning anatomy and imaging reasoning than the PGY3&4 residents (PGY1 vs. PGY3&4. Anatomy learning: mean score = 4.65 vs 3.78, *p* < 0.001; imaging reasoning learning: mean score = 4.53 vs. 3.81, p < 0.001). When asked about their personal attitude toward these training requirements, the residents in the different training years responded similarly, and the mean scores were all close to 4 points or higher. The entire multiple-choice questionnaire presented acceptable reliability and validity, with a Cronbach’s alpha of 0.753 and a KMO of 0.710, *p* < 0.001. In addition, the feedback for the question related to “Learning approaches” revealed that atlas of anatomy and textbooks were the major approaches for the residents to study anatomy, accounting for 85.9% and 38% respectively, and other approaches were relatively fewer taken (21.1% for videos, 11.3% for reference and 5.6% for lectures, and no one used 3D forms).

## Discussion

How to improve the core competence of residents in a more efficient manner is always a standing question for standardized surgical training programs. Surgical education is built on four pillars: surgical theory, clinical surgery, operative techniques and clinical research [12]. Anatomy, the foundation of surgical theory and operative technique, is informative and complex, and it is often the bottleneck of surgery training. In addition, global curriculum reform has led to a reduction in the time and content of anatomical teaching [12, 13]. This requires us to explore more efficient informational-transfer methods such as 3D technology.

Currently, 3D simulation software is used for surgical education and training, such as learning procedures of laparoscopic pyeloplasty via 3D printing and training orthopaedic residents to drill lateral mass screws via 3D navigation [14, 15]. Although the 3D technology applied in medical education has achieved initial success, it is still not systematically used in surgical residency programs. There were some reports of randomized studies regarding the effect of 2D vs 3D teaching modality in the residency. For instance, using 3D printed models of congenital cardiovascular lesions to supplement an educational lecture could improve paediatric medicine residents’ scores on a board-style examination [16]; 3D CT may help learning acetabular fracture patterns and correctly applying a widely accepted fracture classification system among both orthopaedic residents and fellows, compared with 2D axial CT [17]. However, very few studies showed the different attitude, such as Lim’s study who compared the role of XR, CT and 3D models in the accuracy of acetabular fracture identification among orthopaedic residents and concluded 3D models could be an important adjunct to orthopedic residency education but was not significantly superior to identification with CT scans [18]. Additionally, few studies have explored the appropriate introduction time of 3D technology to residency programs; thus, we designed this study to explore the optimal timing for introducing 3D technology into a surgical residency program to improve the trainees’ clinical thinking and learning curve.

In both the 2D and 3D groups, the PGY3&4 residents performed significantly better than the PGY1 residents, and the questionnaire results suggested that it was more difficult for PGY1 residents to learn anatomy and imaging. These results are consistent with the medical learning law that clinical thinking gradually improves as experience accumulates. Nevertheless, the PGY3&4 residents in the 3D group did not show differences from those in the 2D group in the total scores and time spent, which may have been because senior residents had established substantial clinical thinking and spatial imagination. Moreover, different results were observed in the PGY1 residents between the groups. The PGY1 residents in the 3D group performed significantly better in accuracy and rapidity, and this discrepancy underscored the advantages of 3D images for improving intuition and simplifying complex structures. For beginners at the early stage of the learning curve, such as the PGY1 residents, their clinical thinking is still dependent on informational-transfer methods. Thus, 3D images likely had a superior ability to transfer information among the junior residents.

**Fig. 3:**
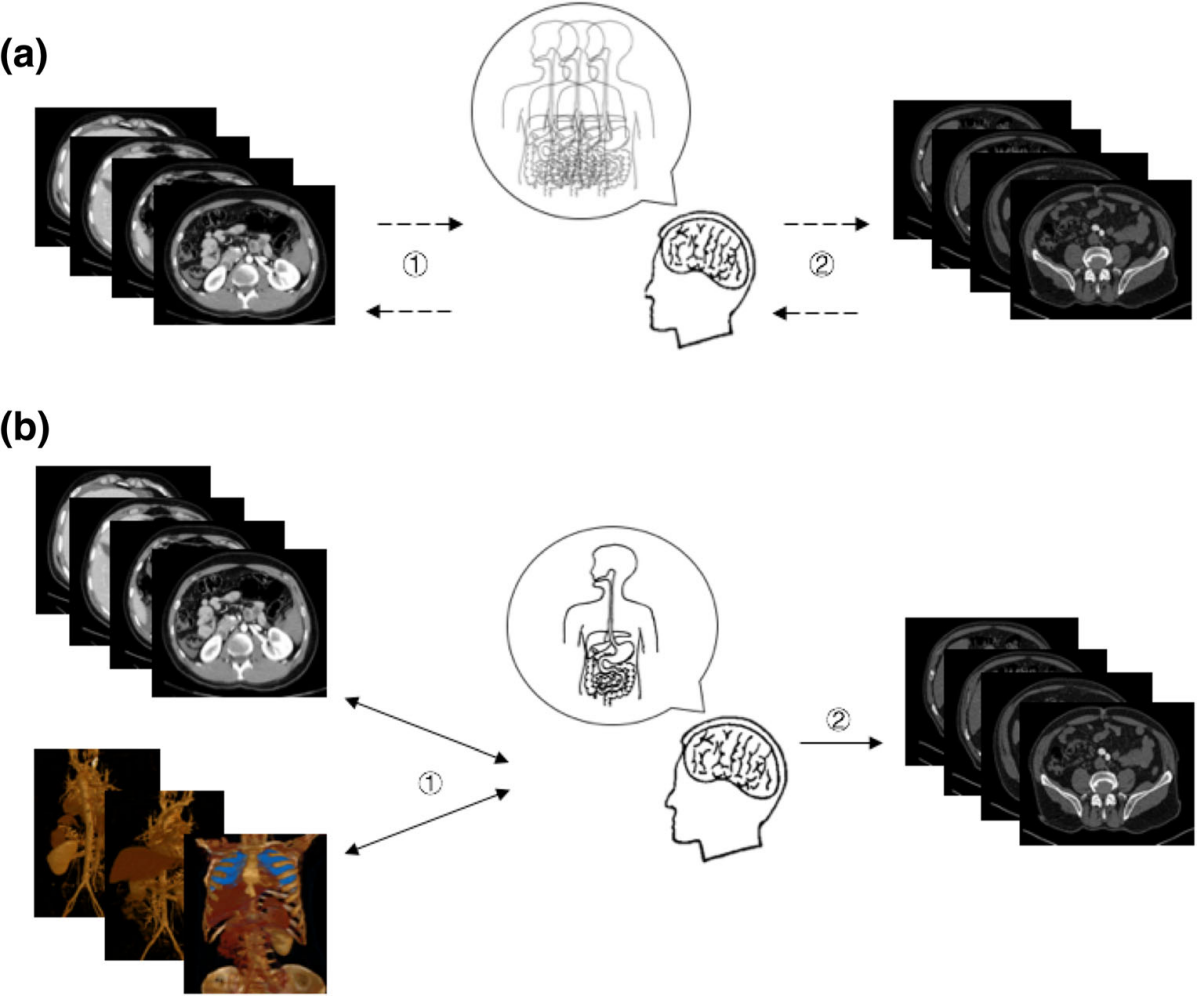
Imaging learning model. **a** Classical learning model: *“2D → 3D → 2D”.* ① Long and difficult learning process that results in vague 3D structures due to lack of immediate correction. ② Process of clinical practice in which residents must correct and rebuild this structure. **b** New learning model combining 2D and 3D images: *“2D + 3D → 3D → 2D”*. ① Accelerated learning process in which structures can be corrected in both a timely and repeated manner, which results in accurate 3D structures. ② Process of clinical practice in which the remembered 3D structures are successfully applied

In the traditional learning model, which could be summarized as “2D → 3D → 2D” (Fig. 3a), trainees must imagine 3D structures with 2D cross-sectional images. This process is long and difficult and often results in the memorization of inaccurate or even incorrect 3D structures because of the lack of an ability to immediately correct these representations. As a result, junior residents will face many problems in the clinic when only 2D images are available. Our findings suggest that junior trainees may be able to interpret 3D images more easily than 2D images. Accordingly, 3D images might be superior in assisting junior residents in establishing 3D models. This new learning model combines 3D and 2D images and might accelerate the learning process by improving the accuracy of 3D structures and deepening the memory of anatomy. We summarize this learning model as “2D + 3D → 3D → 2D” (Fig. 3b). Therefore, we speculate that if this new learning model is systematically introduced into the resident training at PGY1, it may improve the learning curve with respect to the anatomy-imaging-surgery system.

Fewer residents considered anatomy and imaging to be difficult as the grade increased. Although anatomy and imaging did not appear to be difficult for senior residents, most of them agreed that it could strengthen their systematic training. This result revealed the importance of anatomy and imaging for surgeons and indicated that a gap occurs between the urgent demand for training and the current insufficiency in training. In addition, 3D images, which present advantages that include improved intuition and simplified anatomy, are considered to increase the trainees’ interest in anatomy and imaging learning. Moreover, almost all the residents strongly supported the introduction of 3D training into the early stages of residency training.

In addition, residents in our study also encouraged us to introduce CBL into 3D training for anatomy and imaging. Systematic training with real clinical cases can aid in memorizing and understanding material and reduce the gap between theory and practice.

The study limitations are as follows. Although this is a prospective randomized study, a limited number of participants with few female residents were enrolled in this study. To achieve more accurate results, further studies with larger sample sizes and more females enrolled are needed. Besides, although stratified randomization was employed to ensure that residents at a comparable stage of training were assigned to the intervention and control groups, we could not fully account for the possibility that surgical knowledge level and/or hours spent working on surgical cases similar to those assessed in the test may have differed between the participants. Furthermore, as the test used in this study was developed by surgical experts in a single center, the results should be validated with the use of different standardized tests to increase their generalizability. In addition, the impacts of 3D technologies observed inspired us to design novel standard training courses, in which the combined 2D and 3D technology would be introduced, to further confirm the hypothesis raised in this article.

## Conclusion

This randomized study revealed that 3D images improved junior surgical residents’ imaging reasoning performance and were widely accepted and appreciated by all the residents, demonstrating their potential in establishing a more accurate anatomy-imaging-surgery system among young trainees in a more efficient manner. Therefore, it is recommended that combined 3D and 2D training, especially with real clinical cases, be systematically introduced into surgical residency programs early during training.

## Electronic supplementary material

ESM 1 (DOCX 6492 kb)

## Data Availability

The datasets generated during and/or analysed during the current study are available from the corresponding author on reasonable request.
