# Supplementary material for: When to Introduce Three-Dimensional Visualization Technology into Surgical Residency: A Randomized Controlled Trial
Source: J Med Syst. 2019 Feb 9;43(3):71. doi: 10.1007/s10916-019-1157-0 (PMC6373307; doi:10.1007/s10916-019-1157-0)
Supplement: Supplementary file 1 — (DOCX 6492 kb) [file 10916_2019_1157_MOESM1_ESM.docx]

**Supplementary file**

**Imaging Test**

Directions: Questions A1-9 (with 3D reconstructed images) are designed for the 3D group, and questions B1-9 (with 2D CT images) are designed for the 2D group.

| **No.** | **Questions and images** | **Key points of answers** |
| --- | --- | --- |
| **A1** | A 65y male had an annual physical examination. The reconstruction images of his pelvic and abdominal contrast-enhanced CT were shown below. Describe the lesions and make a preliminary diagnosis.  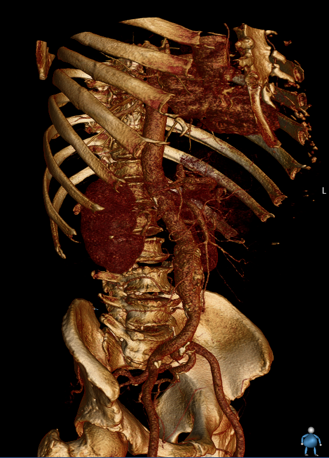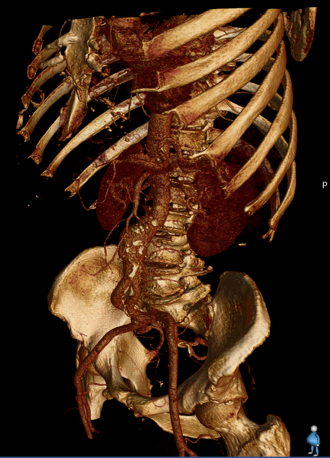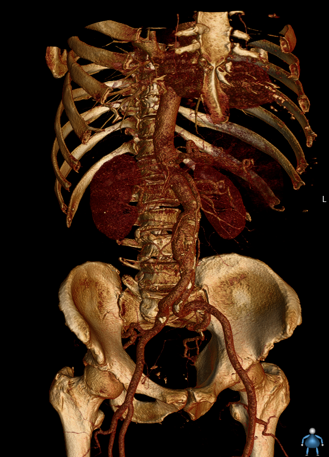 | Description (3*1’):  ①vascular dilations  ②not involving common iliac artery  ③not involving bilateral renal artery  Preliminary diagnosis (1*1’):  ④abdominal aortic aneurysm |
| **A2** | 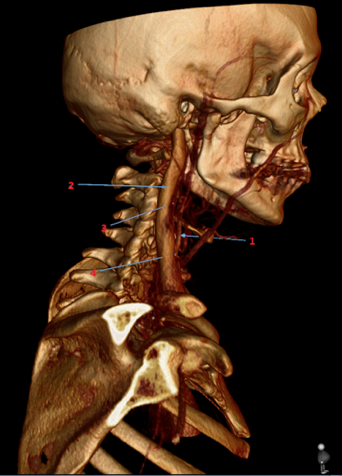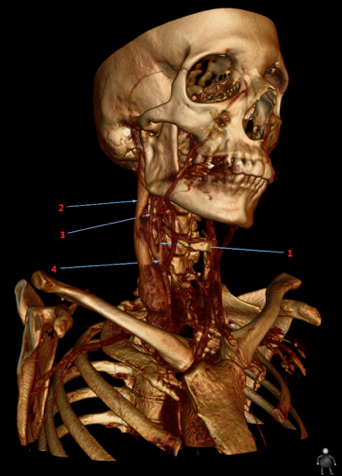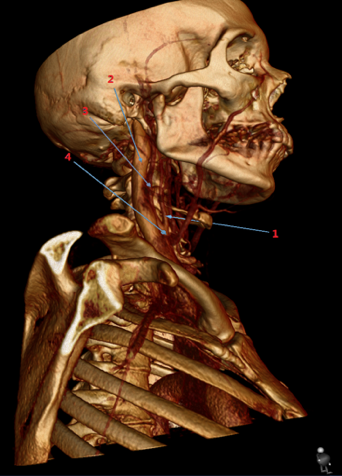The following figures are 3D reconstruction images of the head and neck vessels in different positions. Write the anatomy terms of the vessels marked. | (4*1’)  ①external carotid artery  ②internal jugular vein  ③internal carotid artery  ④common carotid artery |
| **A3** | 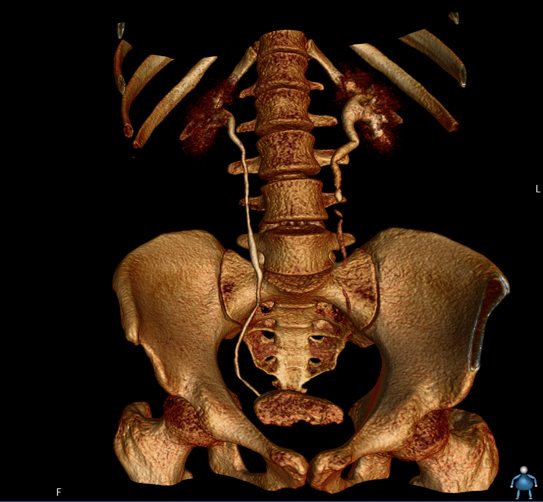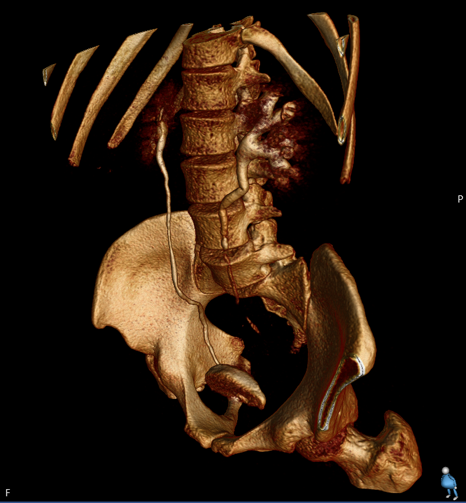A 60y male had gross hematuria. The 3D reconstructed images of CT urography were shown below. Describe the lesions and make a preliminary diagnosis. | Description (3*1’):  ①in the left ureter  ②dilatation of the upper ureter and no appearance of the lower ureter (discontinuity/interruption of ureter)  ③left hydronephrosis  Preliminary diagnosis (1*1’):  ④ureteral occupancy |
| **A4** | 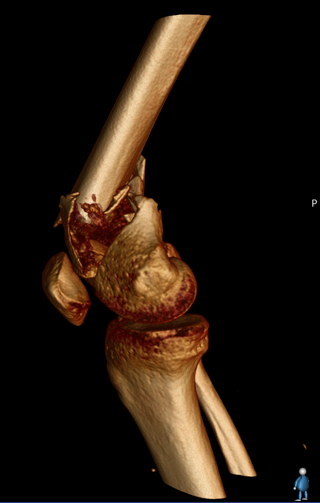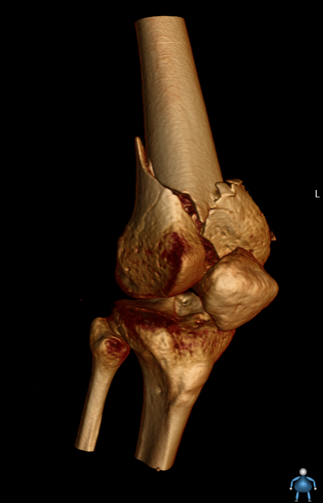A 56y female injured her right leg. The 3D reconstruction images of her knee were shown below. Describe the lesions and make a preliminary diagnosis. | Description (3*1’):  ①at the femoral condyle  ②multiple  ③cortical disruptions  Preliminary diagnosis (1*1’):  ④comminuted femoral intercondylar fracture |
| **A5** | 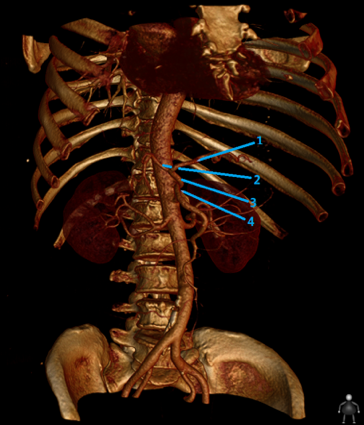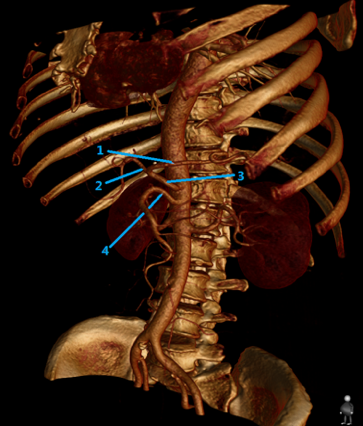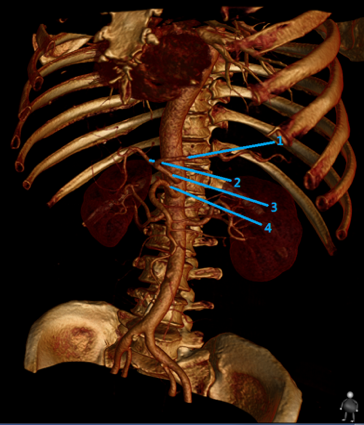The following figures are 3D reconstruction images of the abdominal vessels in different positions. Write the anatomy terms of the vessels marked. | (4*1’)  ①splenic artery  ②common hepatic artery  ③celiac trunk  ④superior mesenteric artery |
| **A6** | 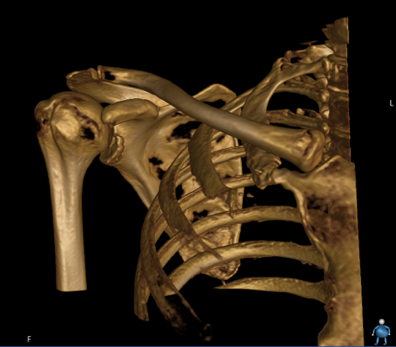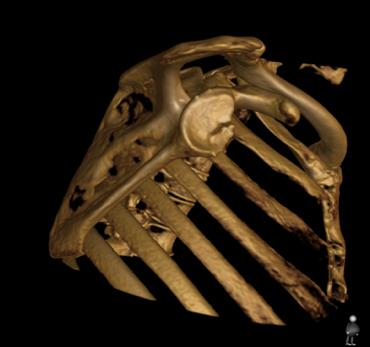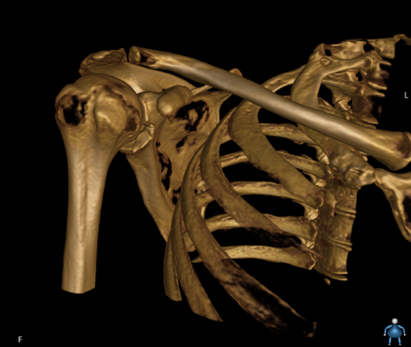A 55y female fell on her right hand, and suffered pain from the right shoulder, but there was no obvious abnormality on the X-ray images. One week later, the pain got worse and she noticed her swelling axillary. The 3D reconstructed images were shown below. Describe the lesions and make a preliminary diagnosis. | Description (2*1’):  **①**at the glenoid fossa of scapula  ②a cortical disruption  Preliminary diagnosis (1*1’):  ③the glenoid fossa fracture |
| **A7** | 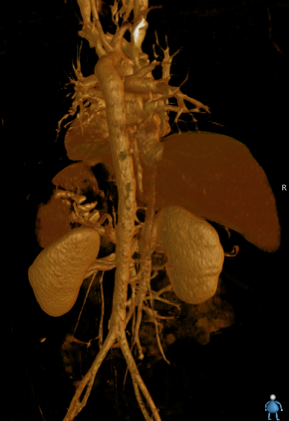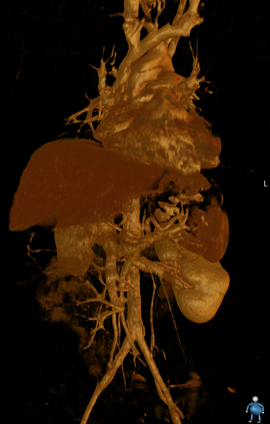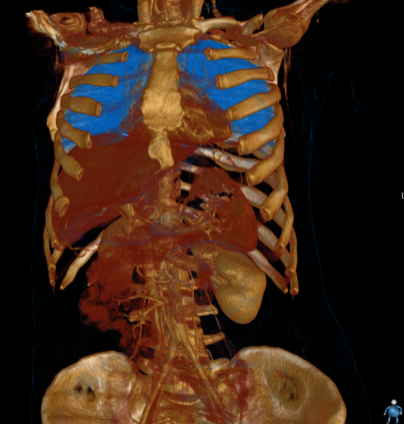A 26y male was hit by a car from the left side while riding, and suffered pain from left lower back. The 3D reconstructed images were shown below. Describe the lesions and make a preliminary diagnosis. | Description (3*1’):  ①at the splenic hilum  ②heterogeneous enhancement  ③incomplete splenic capsule  Preliminary diagnosis (1*1’):  ④splenic rupture |
| **A8** | 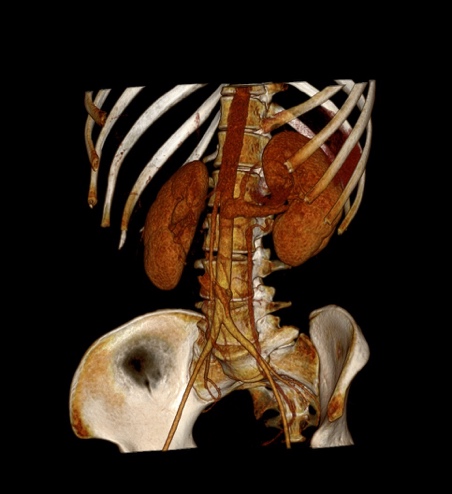A 33y female had paroxysmal hypertension for 5 years. The 3D reconstructed image was shown below. Describe the lesions and make a preliminary diagnosis. | Description (3*1’):  ①near the renal hilum/abdominal aorta  ②a round mass  ③with enhancement / rich blood supply  Preliminary diagnosis (1*1’):  ④pheochromocytoma / paraganglioma |
| **A9** | 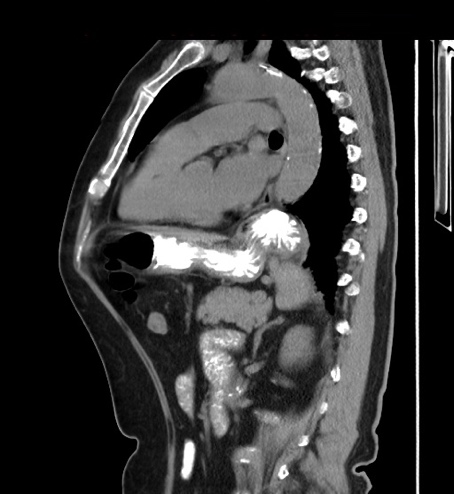A 27y female always had heartburn after a meal. The sagittal reconstruction image of CT, with oral contrast, was shown below. Describe the lesions and make a preliminary diagnosis. | Description (2*1’):  ①the stomach fundus / cardia / part of stomach  ②protruded into the thoracic cavity  Preliminary diagnosis (1*1’):  ③esophageal hiatal hernia |

| **No.** | **Questions and images** | **Key points of answers** |
| --- | --- | --- |
| **B1** | 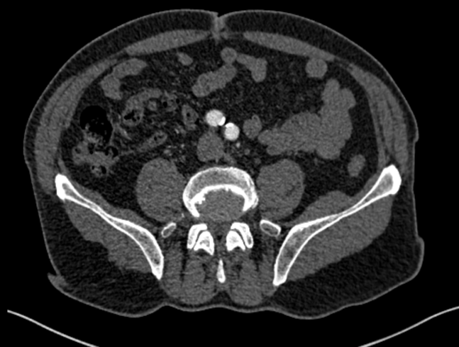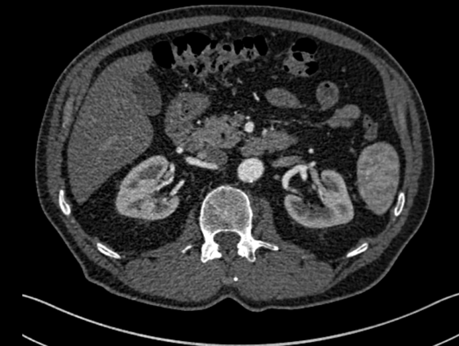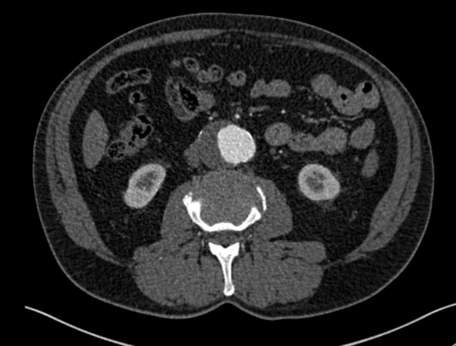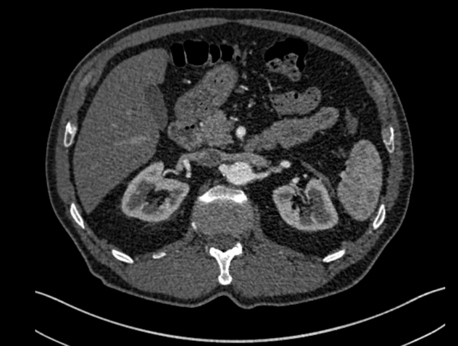A 65y male had an annual physical examination. The contrast-enhanced CT images of his abdomen and pelvic were shown below. Describe the lesions and make a preliminary diagnosis. | Description (3*1’):  ①vascular dilations  ②not involving common iliac artery  ③not involving bilateral renal artery  Preliminary diagnosis (1*1’):  ④abdominal aortic aneurysm |
| **B2** | 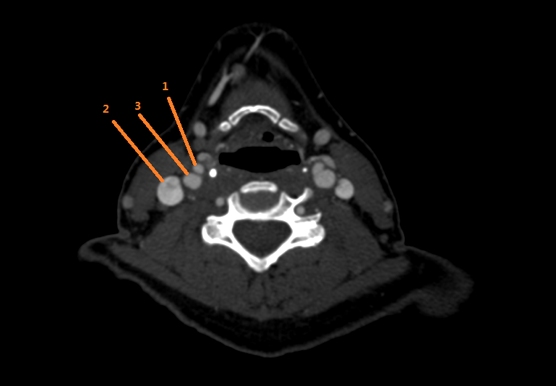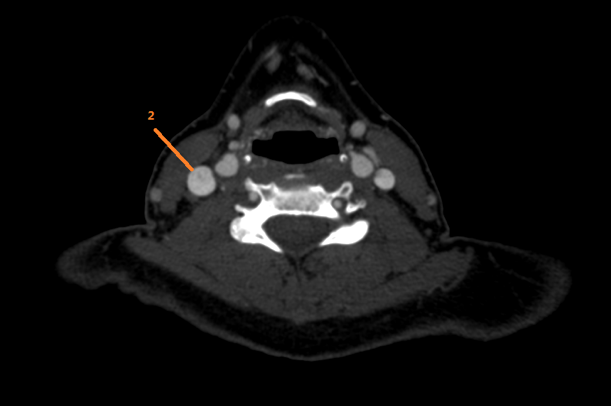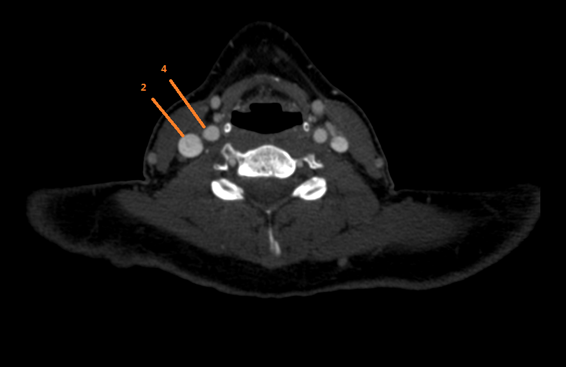The following are contrast-enhanced CT images of head and neck area. Write the anatomy terms of the vessels marked. | (4*1’)  ①external carotid artery  ②internal jugular vein  ③internal carotid artery  ④common carotid artery |
| **B3** | 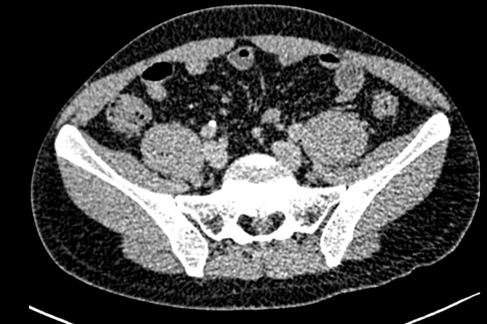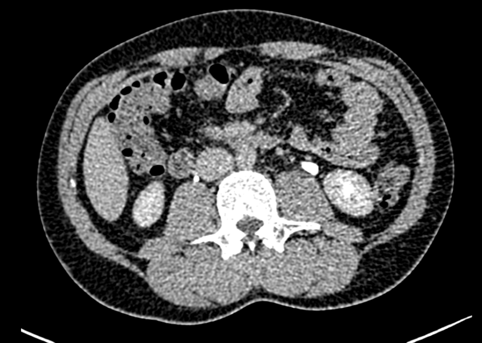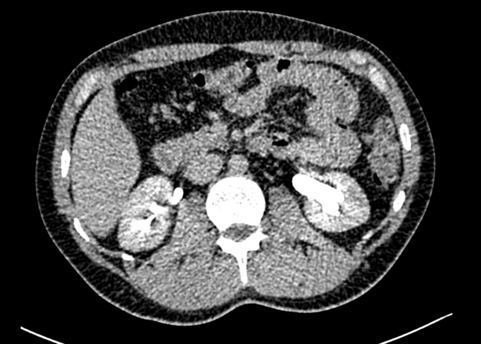A 60y male had gross hematuria. The contrast-enhanced CT images of his abdomen and pelvis were shown below. Describe the lesions and make a preliminary diagnosis | Description (3*1’):  ①in the left ureter  ②dilatation of the upper ureter and no appearance of the lower ureter (discontinuity/interruption of ureter)  ③left hydronephrosis  Preliminary diagnosis (1*1’):  ④ureteral occupancy |
| **B4** | 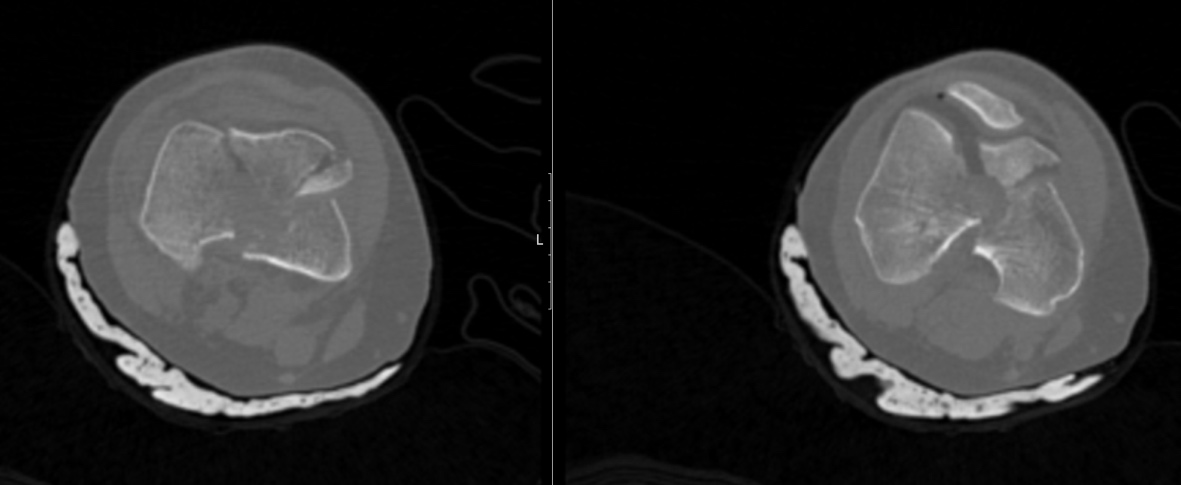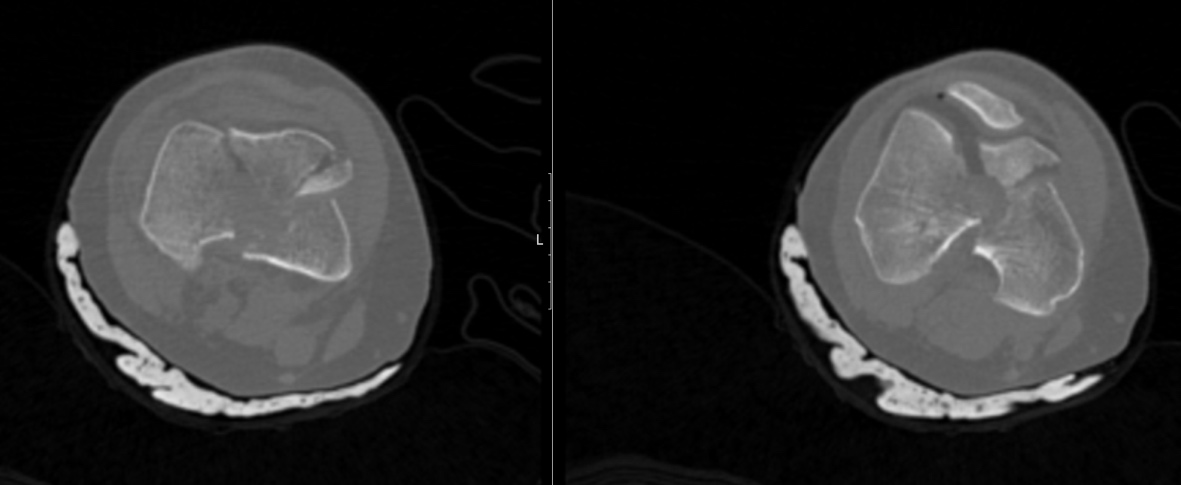A 56y female injured her right leg. The consecutive CT images of her knee were shown below. Describe the lesions and make a preliminary diagnosis. | Description (3*1’):  ①at the femoral condyle  ②multiple  ③cortical disruptions  Preliminary diagnosis (1*1’):  ④comminuted femoral intercondylar fracture |

| **B5** | 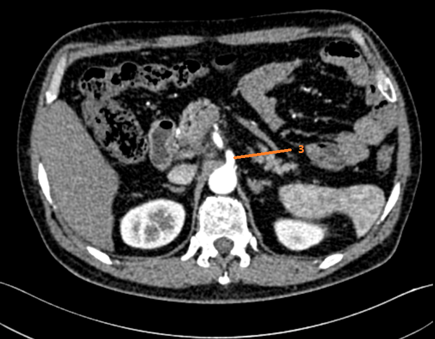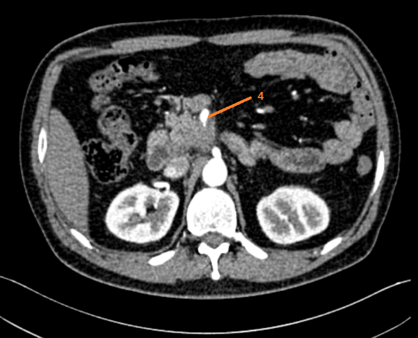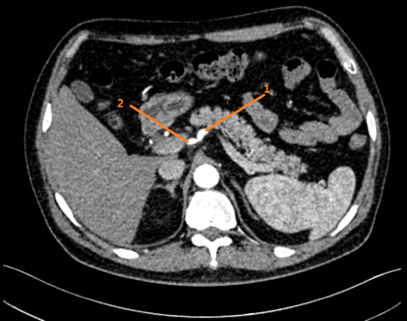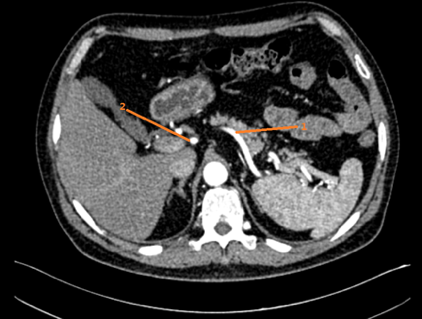The following figures are consecutive contrast-enhanced CT images of the abdomen. Write the anatomy terms of the vessels marked. | (4*1’)  ①splenic artery  ②common hepatic artery  ③celiac trunk  ④superior mesenteric artery |
| --- | --- | --- |
| **B6** | 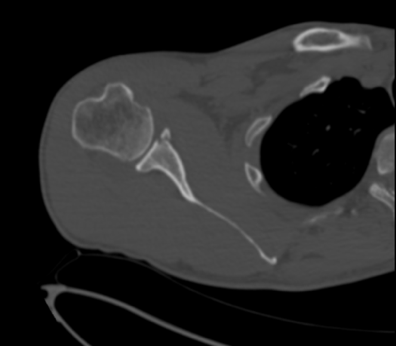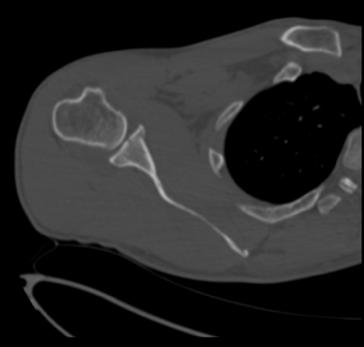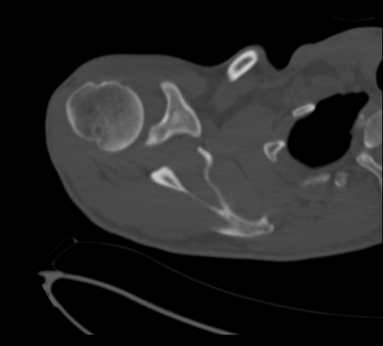A 55y female fell on her right hand, and suffered pain from the right shoulder, but there was no obvious abnormality on the X-ray images. One week later, the pain got worse and she noticed her swelling axillary. The CT images were shown below. Describe the lesions and make a preliminary diagnosis. | Description (2*1’):  **①**at the glenoid fossa of scapula  ②a cortical disruption  Preliminary diagnosis (1*1’):  ③the glenoid fossa fracture |
| **B7** | 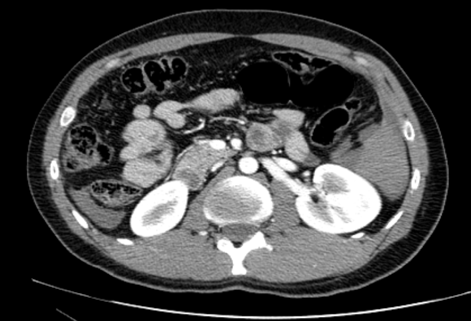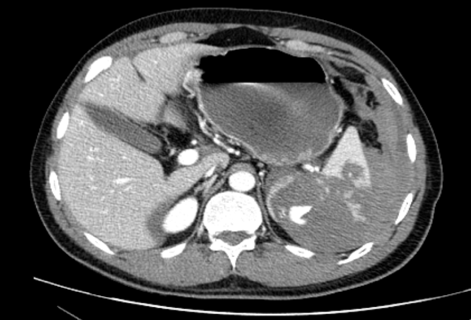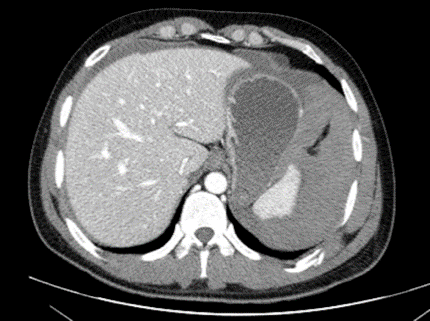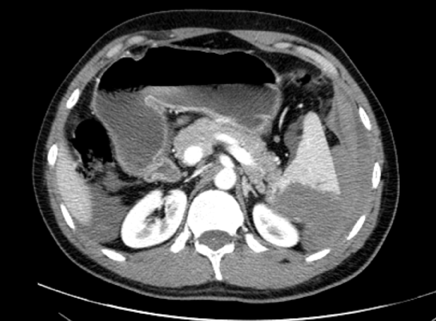A 26y male was hit by a car from the left side while riding, and suffered pain from left lower back. The CT images were shown below. Describe the lesions and make a preliminary diagnosis. | Description (3*1’):  ①at the splenic hilum  ②incomplete splenic capsule  ③perisplenic fluid accumulation  Preliminary diagnosis (1*1’):  ④splenic rupture |
| **B8** | 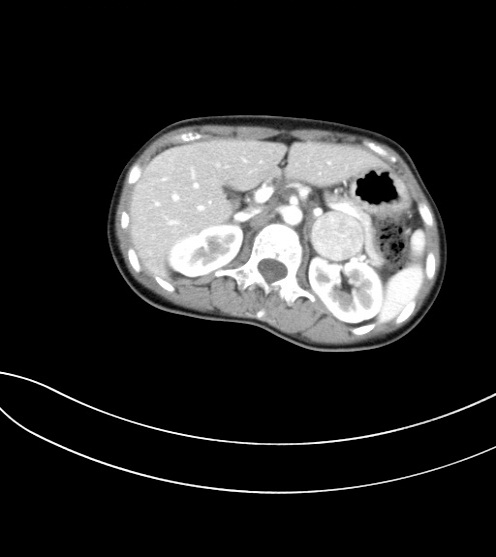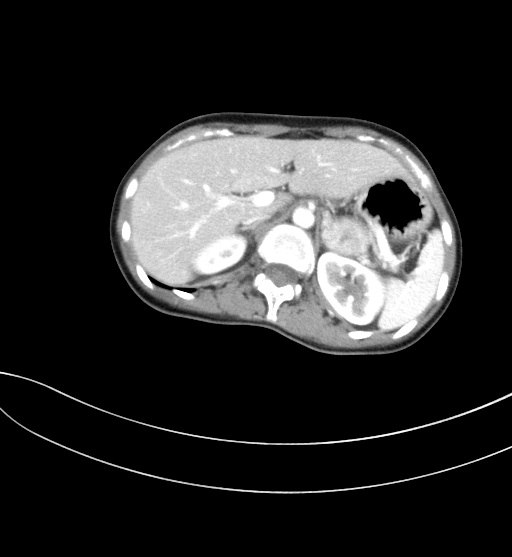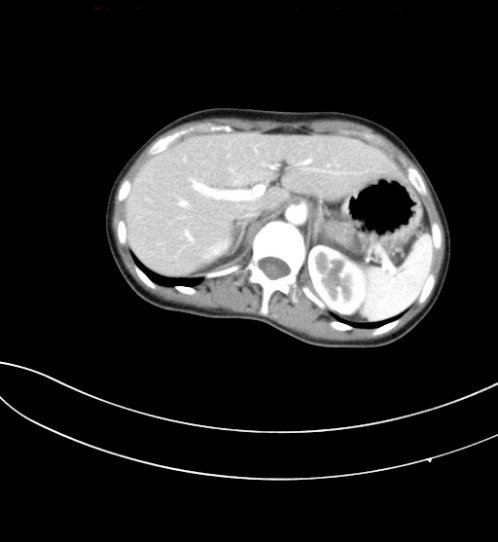A 33y female had paroxysmal hypertension for 5 years. The consecutive contrast-enhanced CT images were shown below. Describe the lesions and make a preliminary diagnosis. | Description (3*1’):  ①near the renal hilum/abdominal aorta  ②a round mass  ③with enhancement/rich blood supply  Preliminary diagnosis (1*1’):  ④pheochromocytoma / paraganglioma |
| **B9** | 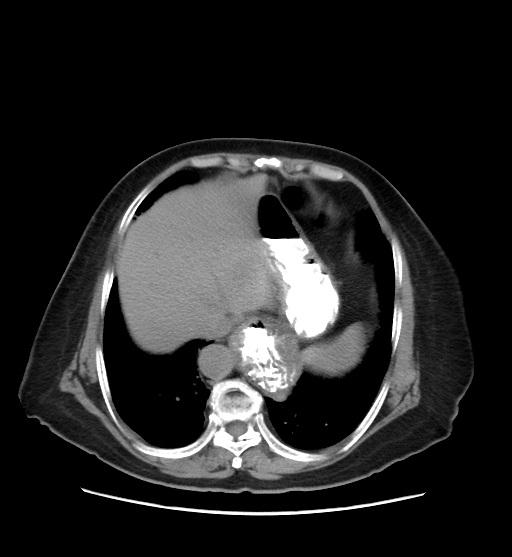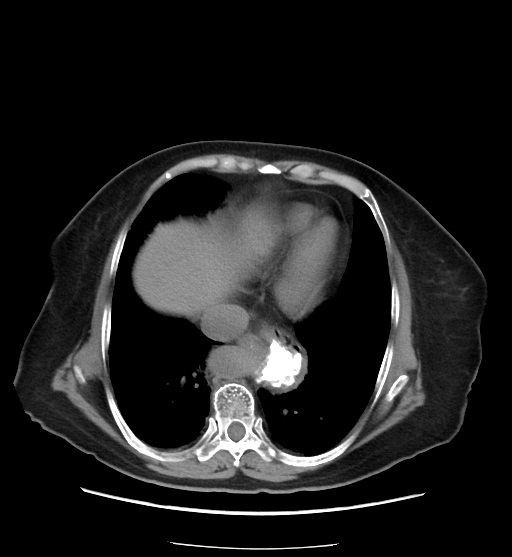A 27y female always had heartburn after a meal. The images of CT, with oral contrast, were shown below. Describe the lesions and make a preliminary diagnosis. | Description (2*1’):  ①the stomach fundus / cardia / part of stomach  ②protruded into the thoracic cavity  Preliminary diagnosis (1*1’):  ③esophageal hiatal hernia |
